# Gestational Diabetes and Long‐Term Risk of Maternal Kidney Disease: Systematic Review and Meta‐Analysis of Population Base Cohort Studies

**DOI:** 10.1002/edm2.70208

**Published:** 2026-04-22

**Authors:** Mansour Bahardoust, Elham Rahimpour, Sheida Shokohyar, Zahra Aghakhani, Ali Delpisheh, Mohammadsadra Shamohammadi, Meisam Haghmoradi, Azin Ghaffari

**Affiliations:** ^1^ Department of Epidemiology, School of Public Health & Safety Shahid Beheshti University of Medical Sciences Tehran Tehran Province Iran; ^2^ Breast Cancer Research Center Iran University of Medical Sciences Tehran Tehran Province Iran; ^3^ Department of Obstetrics and Gynecology Shiraz University of Medical Sciences Shiraz Fars Iran; ^4^ Mass General Hospital Institute of Health Professions Boston Massachusetts USA; ^5^ Cardiovascular Research Laboratory, Spaulding Rehabilitation Hospital, Physical Medicine and Rehabilitation Harvard Medical School Cambridge Massachusetts USA; ^6^ Science York University Toronto Ontario Canada; ^7^ Gastrointestinal and Liver Diseases Research Center Iran University of Medical Sciences Tehran Tehran Province Iran; ^8^ Urmia University of Medical Sciences Urmia West Azerbaizan Iran; ^9^ Rajaie Cardiovascular Medical and Research Center Iran University of Medical Sciences Tehran Tehran Province Iran

**Keywords:** chronic kidney disease, gestational diabetes, maternal, renal outcomes

## Abstract

**Background:**

Gestational diabetes mellitus (GDM) may increase the risk of maternal chronic kidney disease (CKD). The association of GDM with maternal CKD has been heterogeneous across studies, and this association remains controversial. The aim of this systematic review was to investigate the association of GDM with the risk of maternal CKD.

**Methods:**

MEDLINE/PubMed, EMBASE, Scopus and Web of Science were searched, with no time limit, up to 25 August 2025 by two independent investigators to identify studies that had assessed the association of GDM with maternal risk of CKD. Heterogeneity between studies was assessed using Cochrane's Q and I2 tests. Meta‐regression was performed to identify factors associated with heterogeneity.

**Results:**

Eleven cohort studies involving 21,313,434 participants (1,530,599 (7.2%) GDM) were included. Pooled estimates from eleven studies showed that GDM was significantly associated with an increased risk of maternal CKD (HR: 2.19; 95% CI: 1.7, 2.68; p: 0.001, I^2^:92.2%). Further analyses restricted to studies adjusting for key confounders (HR: 2.47; 95% CI: 1.87, 3.08; p: 0.001, I2:24.2%) also showed a significant association. While pooled estimates from three studies did not show a significant association between GDM and an increased risk of AKI (HR: 1.1; 95% CI: 0.94, 1.26). Subgroup analyses showed that GDM was significantly associated with an increased risk of maternal CKD in both DM + (HR: 6.24) and DM ‐ (HR: 1.4).

**Conclusion:**

Gestational diabetes mellitus (GDM) with and without DM was significantly associated with an increased risk of maternal CKD. GDM with DM had a synergistic effect on maternal CKD risk. Although GDM was not significantly associated with increased AKI, only three studies were included in the AKI analysis, which may have affected these results by random error and therefore lack sufficient power and evidence to draw conclusions.

## Graphical abstract

GDM with and without DM was significantly associated with an increased risk of maternal CKD. GDM with DM had a synergistic effect on maternal CKD risk. Women who experience GDM during their pregnancy, especially those who later develop diabetes, should be well informed about their long‐term risk of kidney disease.

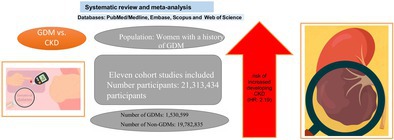

## 1 Introduction

Gestational diabetes mellitus (GDM) is one of the most common pregnancy complications worldwide, with an estimated global prevalence of about 14% [1]. A history of GDM elevates the long‐term risk of metabolic disorders, especially progression to type 2 diabetes mellitus (T2DM) and related diseases [2, 3, 4]. In fact, a history of GDM increases the risk of developing T2DM, along with significantly higher odds of hypertension and cardiovascular disease in the years following pregnancy. These cardiometabolic sequelae are well established [5, 6].

There is substantial biological plausibility that a history of GDM predisposes women to chronic kidney disease (CKD) in the future [7, 8]. According to KDIGO, CKD is defined as persistent abnormalities in kidney structure or function present for at least 3 months, with associated implications for health, and is classified by cause, glomerular filtration rate (GFR) category, and albuminuria [9]. GDM‐related hyperglycemia is associated with dyslipidemia, endothelial dysfunction, and microvascular abnormalities similar to those seen in prediabetes, and these vascular changes may involve the renal microcirculation [8, 10, 11]. Women who had GDM are known to have long‐term impairments in vascular endothelial function, which therefore increase the risk of kidney injury even if T2DM does not develop [12, 13]. Also, there has been an association of GDM with early changes in renal function, including elevated estimated GFR indicative of glomerular hyperfiltration [14]. These pathways, direct injury to the endothelium and indirect effects via T2DM and hypertension, support the history of prior GDM leads to long‐term maternal CKD risk [15]. Despite strong biological plausibility, the literature linking GDM to maternal CKD later in life is inconsistent and hampered by significant methodological issues [8, 16].

Recent studies have applied heterogeneous definitions of both GDM and CKD, inconsistently adjusted for confounding, and frequently lacked adequate follow‐up to capture long‐term outcomes [7, 15, 17, 18, 19, 20]. To address these limitations, we conduct a systematic review and meta‐analysis of observational studies to quantify the association between history of GDM and subsequent maternal CKD.

## 2 Methods

### 2.1 Protocol Registration and Search for Potential Studies

This systematic review and meta‐analysis was conducted after determining the research question and registering its protocol in PROSPERO (international systematic review registry) based on the PRISMA (Preferred Reporting Items for Systematic Reviews and Meta‐Analyses) guidelines [21].

### 2.2 Literature Search

Databases, including MEDLINE/PubMed, EMBASE, Scopus, and Web of Science, were searched without time limit up to August 25, 2025, by two independent investigators using mesh terms to identify studies that assessed the association of GDM with the risk of developing maternal CKD. Also, the sources of the studies were imported, and Google Scholar was reviewed as grey sources.

The general search strategy was determined based on PICO (Patient, Intervention, Comparison, and Outcome) formula.

Based on the research question, we defined PICO as follows: Population: Women with a history of GDM, Intervention/Comparison: GDM vs. no GDM, Outcomes: Risk of developing any maternal CKD postpartum and AKI.

The databases were searched using a combination of the following keywords: Gestational diabetes; Gestational Diabetes Mellitus, Pregnancy‐Induced Diabetes, renal outcome, chronic kidney disease, Maternal. The search strategy for the databases is reported separately (Table S1).

Acute renal failure and acute tubulointerstitial nephritis were defined as AKI in original studies. Any kidney disease caused by hypertension, chronic tubulointerstitial nephritis, and glomerular disease, and proteinuria confirmed by ICD codes were defined as CKD [22] (Table S2).

### 2.3 Inclusion and Exclusion Criteria

Inclusion criteria for this systematic review included observational studies (cohorts and cross‐sectional) that assessed the association of GDM with the risk of developing maternal CKD in women with at least one pregnancy; observational studies with at least 1 year of postpartum follow‐up; studies that reported the effect size of GDM on maternal CKD risk regardless of DM status; cohort studies with at least 100 patients in the GDM subgroup; and studies published in English. Studies that did not report details of the association of GDM with maternal CKD and were limited to comparing the frequency or incidence of maternal CKD, studies that reported the association of adverse pregnancy outcomes with the risk of maternal CKD, and the population of women with GDM were not distinguishable from other complications, lack of access to full text of articles, letters to the editor, animal/laboratory studies, case reports, and randomized controlled clinical trials (RCTs) were defined as exclusion criteria.

### 2.4 Screening

Articles found from the searched databases were aggregated. Using EndNote software, duplicate and shared articles between sources were identified and removed by two independent researchers. The remaining articles were reviewed by two independent researchers for relevance to the research question, including title and, where appropriate, abstract. Any disagreements about whether or not to remove an article were resolved by a third researcher. Inclusion and exclusion criteria were applied.

### 2.5 Data Extraction

To extract data, a literature review was first conducted. The variables required for extraction were determined based on the research question by a committee consisting of nephrologists and epidemiologists. The variables were prepared in the form of checklists in two separate Excel files and provided to two independent researchers. Two independent researchers performed the extraction of variables, and a third researcher resolved any discrepancies. The extracted variables included study authors, study year, geographical area of study, study design, mean age of women, smoking history, type 2 diabetes, total number of study subjects, number of GDM cases, number of women with preeclampsia, mean BMI, median gravidity, dyslipidemia, number of maternal CKD cases, type of CKD, effect size (Odds ratio) of the association of GDM with maternal CKD and AKI at 95% confidence interval, and mean follow‐up period. We contacted the study authors to find missing data.

### 2.6 Quality Assessment

The quality of the cohort studies included in this meta‐analysis was assessed using the Newcastle‐Ottawa Quality Assessment Form for Cohort Studies [23]. The quality of the included studies and the risk of bias are assessed based on this checklist in three key areas: selection, comparability, and outcome. Finally, a score is assigned to each study and the quality of studies is classified into three categories: good, fair, and poor. We also assessed the certainty of evidence using the GRADE approach [24].

### 2.7 Statistical Analysis

Data analysis was performed using Stata version 17 with random effects models. To estimate the incidence of maternal CKD in women with GDM, the meta‐prop command was used at a 95% confidence interval (95% CI). The pooled effect size of the association of GDM with the risk of developing maternal CKD was reported as odds ratio (HR and 95% CI). To assess heterogeneity between studies, we used the Cochran Q and I^2^ tests. I^2^ values were interpreted as follows: 0%–40% may indicate no significant heterogeneity; 40%–75% may indicate moderate heterogeneity; and > 75% indicated significant heterogeneity. Given the presence of heterogeneity between studies to estimate the overall effect size, we used meta‐regression analysis to identify factors associated with heterogeneity. The publication bias was assessed with the Egger test, and the results were visually displayed in a funnel plot. Descriptive results were reported using tables and figures. Sensitivity analyses were performed to examine the impact of individual studies on the overall estimate of the association of GDM with the risk of developing maternal CKD. Subgroup analyses were performed to estimate the effect size of the association of GDM with the risk of developing maternal CKD based on the presence or absence of DM (DM+ or DM‐), and the results were reported separately.

## 3 Results

**FIGURE 1:**
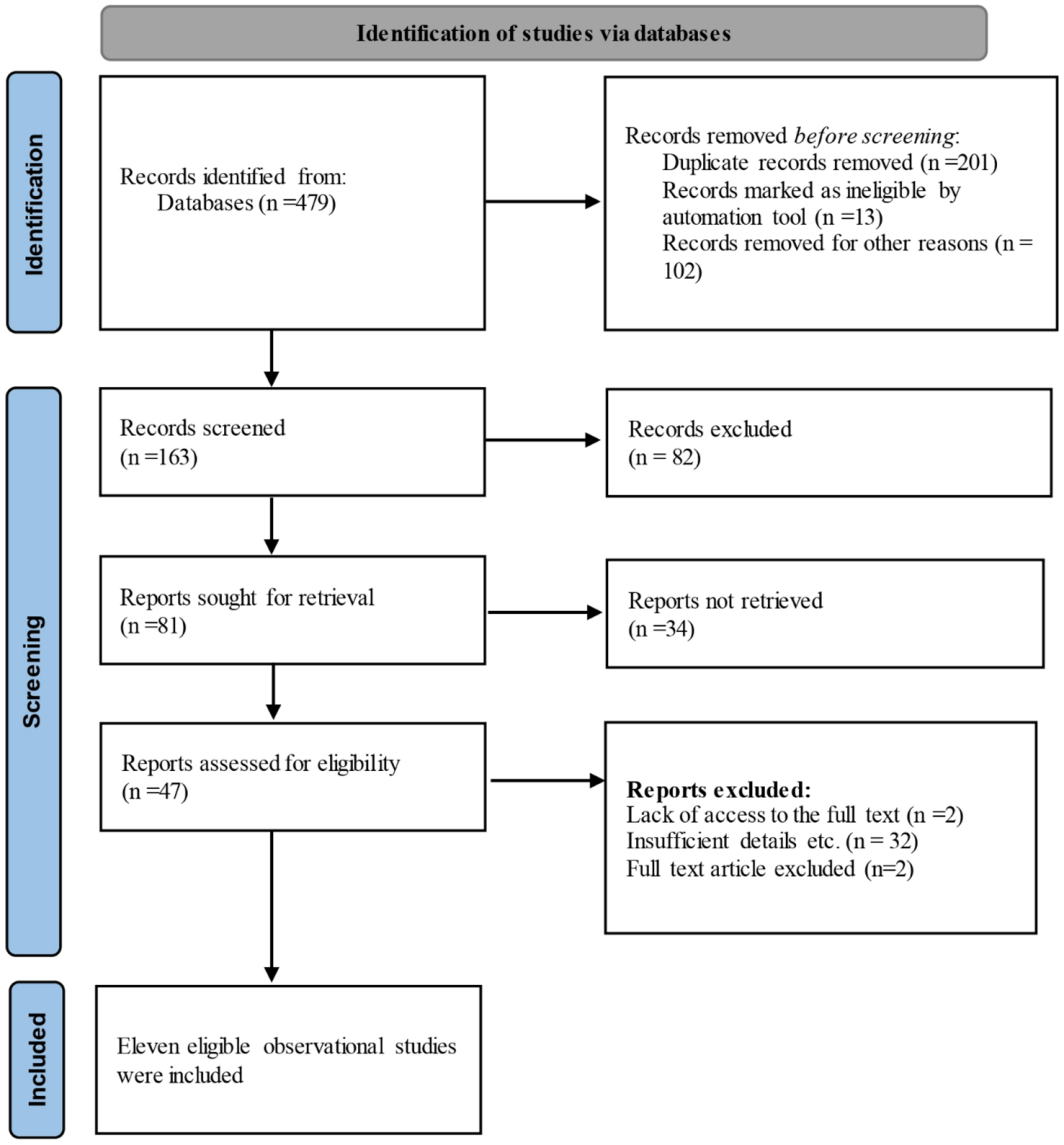
Flowchart page of studies based on PRISMA 2020.

The initial database search yielded 479 relevant articles. 200 and one duplicate articles across databases were removed. 100 and 63 articles were evaluated based on the research question, title and abstract. The full text of 47 articles was read. In this meta‐analysis, 11 population‐based cohort studies [7, 14, 15, 16, 17, 18, 19, 20, 25, 26, 27] including 21,313,434 participants were finally included (Figure 1).

Approximately 7.2% (1530599) of them had GDM. The mean age of mothers with GDM was 31.1 ± 4.8 years. The mean BMI was 26.7 ± 3.1 kg/m^2^. The prevalence of preeclampsia in GDM was 9.6% (4950/51598). Among GDM mothers, approximately 15.2% (216,881/1427993) were simultaneously DM+. Approximately 15.4% (11,740/76171) of GDM mothers were smokers. According to the study quality assessment checklist, the majority of studies had a low risk of bias (9 of 11). The results of the study quality assessment are reported separately for each study (Table S3). In terms of certainty of evidence based on the grading tool, the majority of studies had a high certainty. The mean follow‐up period of patients was 13.8 ± 3.1 years. The demographic and baseline characteristics of the participants in the studies included in this meta‐analysis are reported in Table 1.

**TABLE 1:** Baseline characteristics, certainty of evidence, and risk of bias of studies included in this meta‐analysis.

Authors (Year)	Design	Total population	N of maternal GDM	Age	BMI	HTN	Preeclampsia	DM+	Smoking	N of any CKD	Mean follow up (Year)	CKD sub‐type	Risk of bias	Certainty of evidence
AS Bomback (2010) [17]	Population‐based cohort	25,616	571	51.5	30	354	NA	NA	NA	122	NA	Any CKD	Good	High
O Beharier (2015) [26]	Population‐based cohort	97,968	9542	32.2	26.1	NA	NA	NA	986	23	11	Any renal disease	Good	High
S Rawal (2018) [14]	Population‐based cohort	1226	607	31.6	27.1	91	7	183	158	56	13	Any CKD	Good	High
EW Dehmer (2018) [16]	Population‐based cohort	820	101	25.3	23.5	16	NA	63	39	NA	20.8	Any CKD	Good	Moderate
PM Barrett (2022) [25]	Population‐based cohort	1,121,633	15,595	28.5	26.4	NA	1828	844	2304	NA	12.2	Any CKD	Good	High
ST Tseng (2023) [18]	Population‐based cohort	358,055	71,611	31.06	NA	NA	NA	NA	NA	18	5	Any CKD	Good	High
MJL Hare (2023) [7]	Population‐based cohort	10,508	239	26.6	26.8	154	101	239	320	66	12.1	CKD and ESKD	Fair	High
MH Christensen (2024) [15]	population‐based cohort	697,622	23,710	28.1	27.2	553	1667	97	3760	210	11.5	Any CKD	Good	High
BM Daly (2024) [19]	Population‐based cohort	68,682	11,447	32.3	NA	NA	1347	2769	316	358	NA	Any renal disease	Good	High
C Crump (2024) [27]	Population‐based cohort	667,774	36,269	27.5	26.5	351	NA	6119	3857	413	25	Any CKD	Good	High
A Backal (2025) [20]	Population‐based cohort	18,263,530	1,360,907	29.9	30	354	NA	206,567	NA	361	NA	Any renal disease	Good	High

### 3.1 Incidence of CKD in Mothers With a History of GDM

**FIGURE 2:**
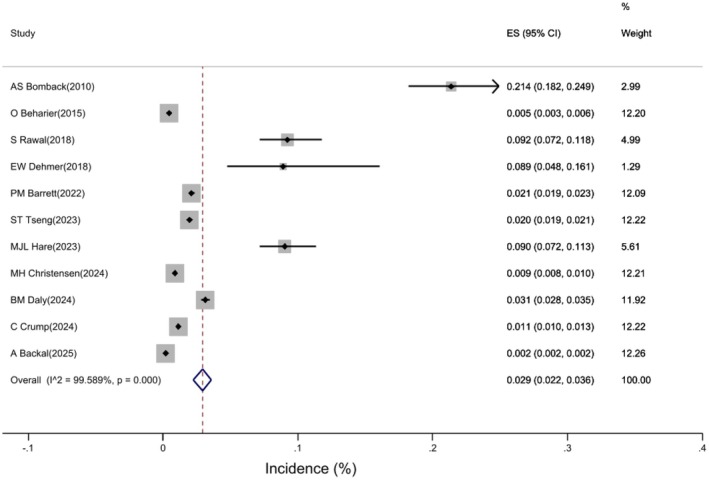
Forest plot of incidence of CKD in mothers with a history of GDM.

Based on a pooled estimate from 11 studies, the pooled incidence of CKD in mothers with a history of GDM was 2.9% (95% CI: 2.2, 3.6%). Figure 2.

### 3.2 Association of GDM With the Risk of Developing Renal Outcomes

**FIGURE 3:**
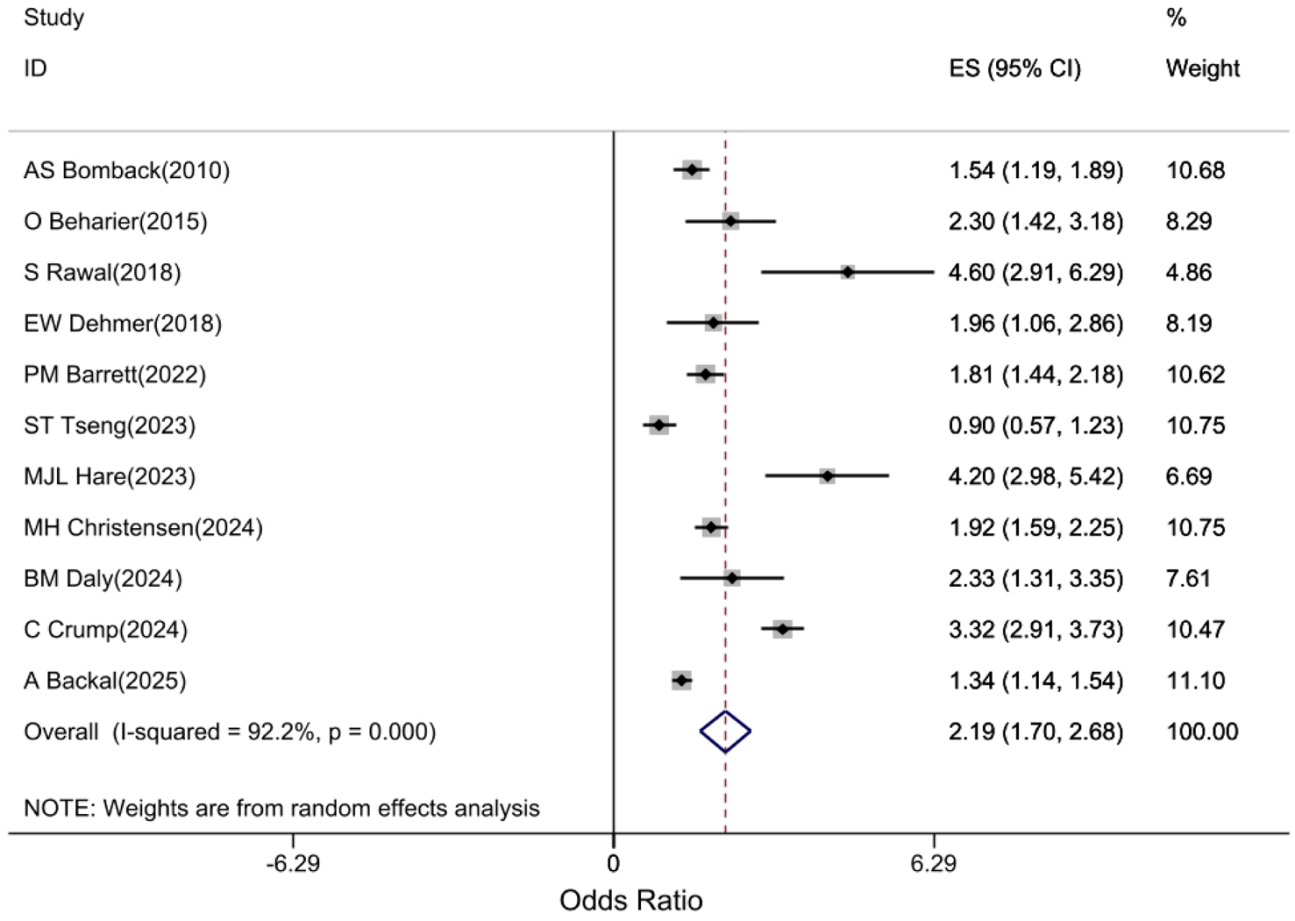
Forest plot of association of GDM with the risk of developing CKD in women.

Pooled estimates from 11 studies [7, 14, 15, 16, 17, 18, 19, 20, 25, 26, 27] showed that GDM was significantly associated with an increased risk of CKD (regardless of subtype) (HR: 2.19; 95% CI: 1.7, 2.68; p: 0.001, I2: 92.2%) (Figure 3).

**FIGURE 4:**
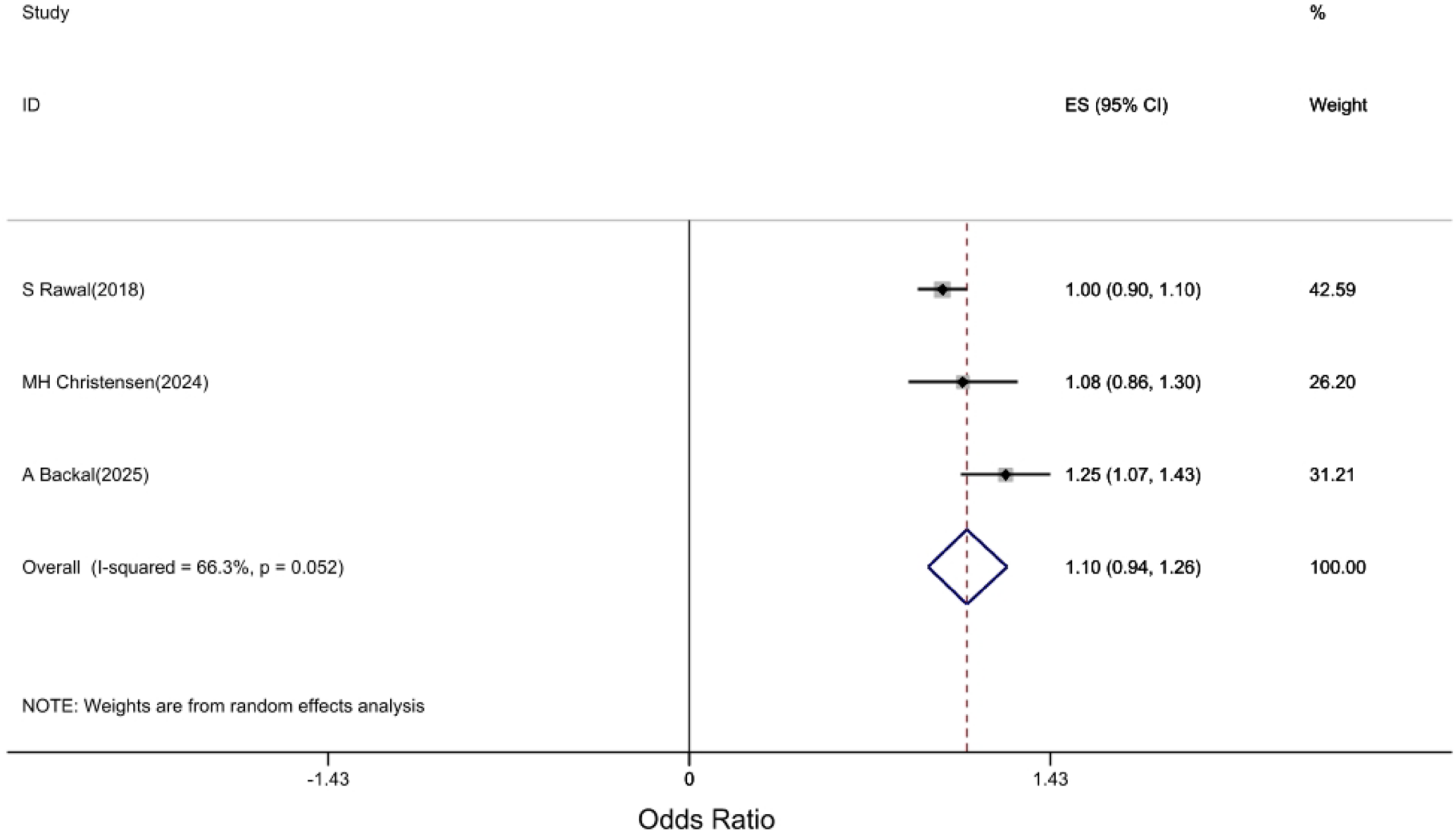
Forest plot of association of GDM with the risk of developing acute renal failure in women.

The association of GDM with the risk of developing acute renal failure was examined in three studies [14, 15, 20]. The pooled estimates showed that GDM was not significantly associated with an increased risk of AKI (HR: 1.1; 95% CI: 0.94, 1.26; p: 0.23, I2: 66.3%) Figure 4.

### 3.3 Sub Group Analysis

**FIGURE 5:**
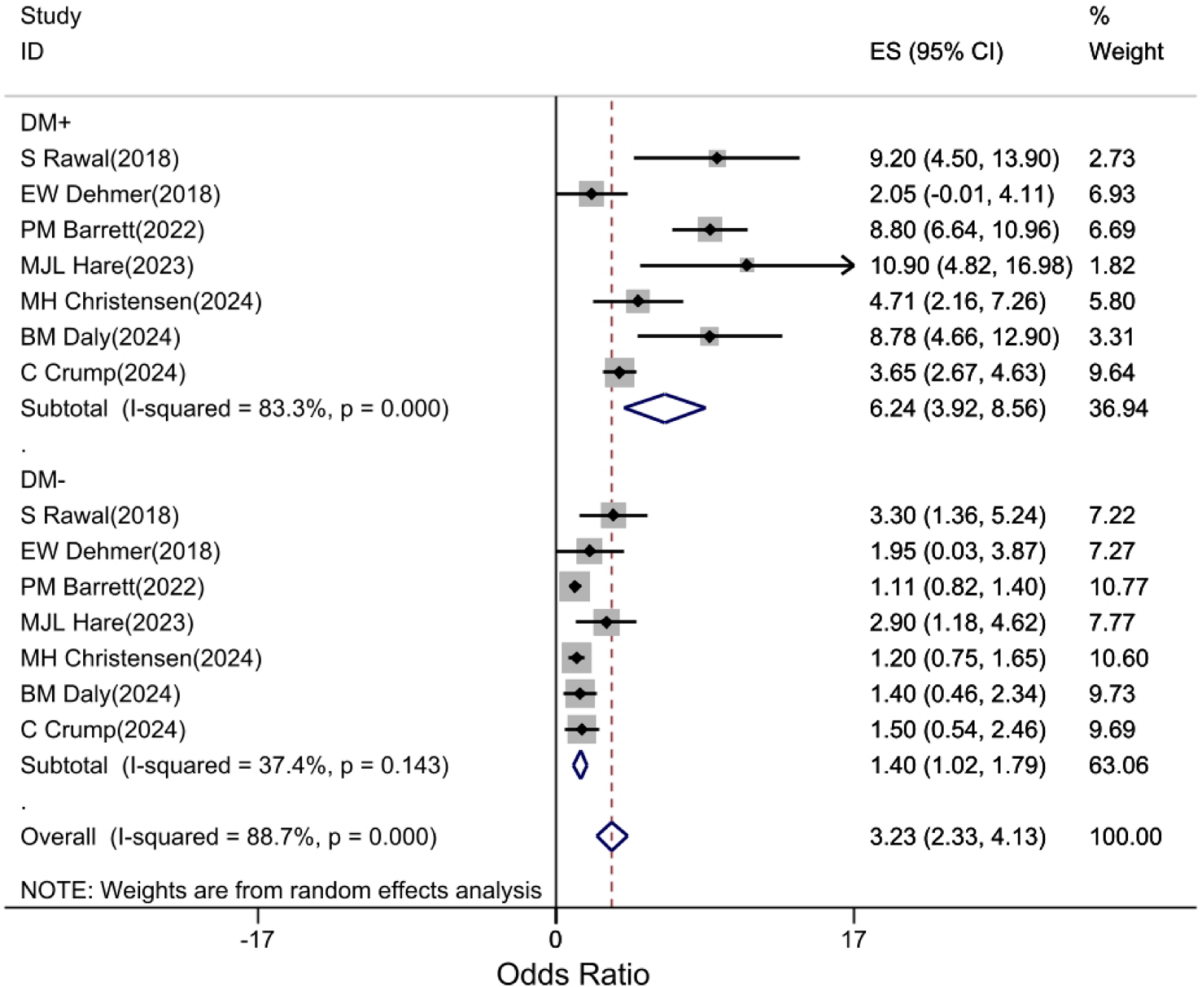
Forest plot of association of GDM with the risk of developing CKD in women based on DM (+ or −).

The association of GDM with the risk of developing any renal outcome was examined in seven studies based on the presence or absence of concomitant DM. Subgroup analyses showed that GDM with and without diabetes was associated with an increased risk of CKD in women. However, DM increased the risk of developing CKD, and the risk of renal outcomes was significantly higher in GDM women with DM+ (HR: 6.24; 95% CI: 3.92, 8.56; p: 0.001) than in DM‐(HR: 1.4; 95% CI: 1.02, 1.79; p: 021) (Figure 5).

### 3.4 Sensitivity Analysis and Meta‐Regression

Sensitivity analysis was performed to estimate the effect size of each study on the overall estimate of GDM on CKD in women after pregnancy. The results of the sensitivity analysis showed that studies with GDM sample sizes greater than 20,000 and studies with follow‐up greater than 10 years had the greatest effect on the overall result (Figure S1). The results of sensitivity analysis based on original data adjusted for baseline variables. Seven studies showed that, in accordance with the overall estimate, in the adjusted state for baseline confounders, GDM was associated with an increased risk of developing CKD(HR: 2.47; 95% CI: 1.87, 3.08; p: 0.001, I2:24.2%).

Meta‐regression results showed that study sample size, number of GDM, CKD definition, mean follow‐up period, DM+, geographical region, and mean age were significantly associated with the association of GDM with the risk of CKD (*p* < 0.05). (Table 2).

**TABLE 2:** Meta‐regression model of the effect of variables on effect size.

Variable	*β*	se	*p*
Sample size	0.42	0.13	0.004
Number of GDM	−0.52	0.15	0.001
Mean age	0.29	0.19	0.001
GDM+	2.56	0.54	0.001
Mean follow‐up period (> 10 Year)	0.56	0.26	0.012
Geographical region	−0.31	0.12	0.029
CKD definition	0.23	0.09	0.025

### 3.5 Publication Bias

**FIGURE 6:**
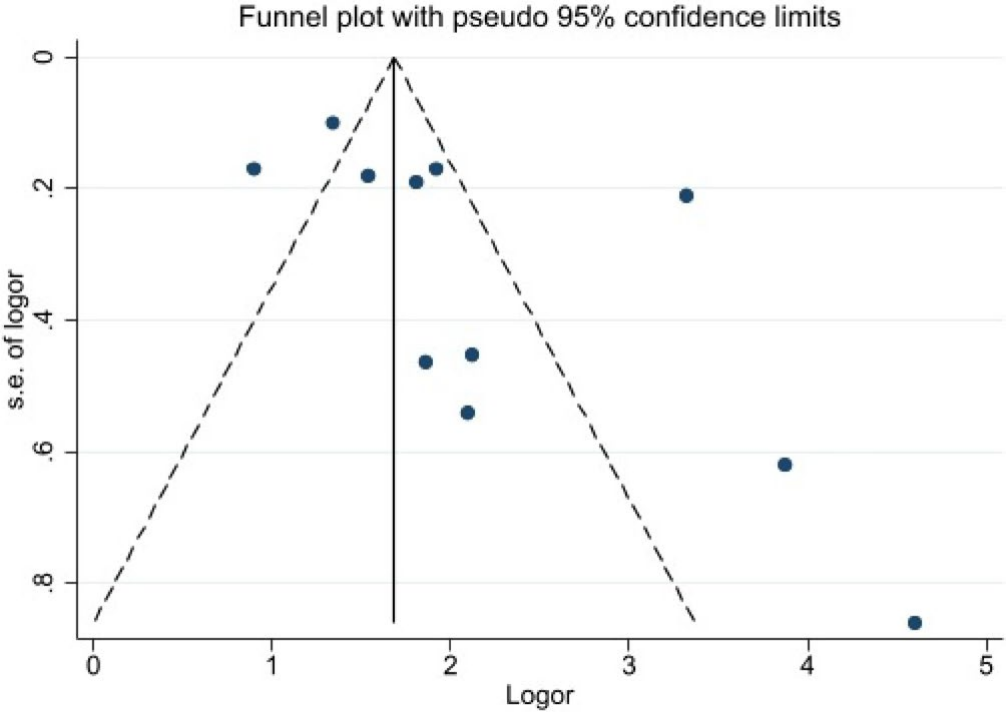
Bias publication assessment in the funnel plot.

The results of the Egger's test showed no significant effect of publication bias on the overall estimate of the association of GDM with the risk of developing renal outcomes (Egger's test: 2.89, 95% CI: −0.59, 6.37, P: 0.075). The distribution of studies by outcome is also reported in a funnel plot (Figure 6).

## 4 Discussion

Gestational diabetes mellitus (GDM), which is only present during pregnancy, can be associated with health outcomes, including vascular and renal outcomes, after subsequent pregnancies. The magnitude of the effect of GDM on the risk of developing CKD and acute renal failure has been heterogeneous across studies, and the association of GDM with renal outcomes remains controversial. In this systematic review and meta‐analysis, we examined the association of GDM with the risk of developing renal outcomes, including CKD and acute renal failure, by pooling the results of 11 studies including 1,530,599 GDM with a mean age of 30 years. We also examined the effect of GDM in the DM + and DM − subgroups.

According to our meta‐analysis, approximately 2.9 out of 100 mothers with GDM will experience maternal CKD postpartum. GDM increased the risk of maternal CKD development by more than 2‐fold (HR: 2.19), regardless of DM presence or absence. Based on the pooled estimates of the three studies, GDM was not significantly associated with an increased risk of developing AKI; however, this lack of association may be due to the limited number of studies conducted in this area, and further studies in this area may change the results. Only three studies were included in the AKI analysis, which may have affected these results by random error and therefore lack sufficient power and evidence to draw conclusions. Prospective studies with larger sample sizes could change these estimates.

The magnitude of the association between GDM and maternal CKD varied by DM status (+ or −). The risk of developing maternal CKD was significantly higher in GDM and DM + mothers (HR: 6.24) than in DM − mothers (HR: 1.4). The co‐occurrence of diabetes with GDM has a positive association and can increase the risk of developing GDM by more than 4 fold compared with DM − individuals.

Based on the study quality assessment tools, the risk of bias was low in the majority of studies. In terms of certainty of evidence, almost all studies had high certainty. The results of the Egger test showed no evidence of publication bias, and publication bias did not significantly affect the estimated results.

The heterogeneity between studies was significant, and we performed meta‐regression to identify factors associated with heterogeneity between studies. The results of meta‐regression analysis showed that a significant part of the heterogeneity between studies could be explained by differences in mean follow‐up, socioeconomic status, race/ethnicity, demographic characteristics of patients including mean age, presence or absence of DM, and differences in geographical regions. Studies have shown that the risk of CKD varies in different geographical regions, which can be influenced by lifestyle, race, and access to health services [28, 29, 30, 31, 32, 33]. Similarly, the prevalence of diabetes, which in our study positively interacted with GDM for the risk of maternal CKD, varies in different geographical regions [29, 34]. EK Tannor et al. [35] demonstrated that low socioeconomic status increases the risk of developing CKD, as well as the mechanisms leading to increased disease burden, faster progression, and significant morbidity and mortality from CKD from fetal life to adulthood. In another study, RE Patzer et al. [36] showed that low socioeconomic status and racial and ethnic disparities were significantly associated with an increased risk of chronic kidney disease, progression to end‐stage renal disease, inadequate dialysis treatment, reduced access to kidney transplantation, and adverse health outcomes, which could be related to heterogeneity between studies.

Age and mean follow‐up period were other factors associated with heterogeneity between studies. Studies have shown that the risk of diabetes and CKD increases with age. In addition, the risk of maternal CKD is higher in studies with longer average follow‐up periods, which may be explained by the progressive nature of the disease and the concomitant increase in maternal age [37, 38, 39].

Although to our knowledge, no meta‐analysis has examined the association of GDM with the risk of developing maternal CKD, limited reviews have suggested that GDM is associated with an increased risk of developing vascular outcomes [8, 40].

In a meta‐analysis by PM Barrett et al. [8] in 2020, assessing the association of adverse pregnancy outcomes with maternal long‐term kidney disease, exposure to adverse pregnancy outcomes, including hypertensive disorders of pregnancy and preterm delivery, was associated with a higher risk of long‐term kidney disease. They also reported that GDM was associated with an increased risk of CKD in black women but not in white women, and the number of studies included in this meta‐analysis was too limited to assess CKD (only two studies). In our comprehensive review, GDM was associated with an increased risk of CKD even in DM‐negative patients. In a narrative review, K Siddiqui et al. [41] qualitatively examined the potential impact of gestational diabetes on long‐term kidney disease. They showed that GDM can be an independent risk factor for CKD, and our study quantitatively confirmed these findings. In a narrative review, R Neiger et al. [42] showed that the risk of developing cardiovascular and metabolic diseases in later life was higher in women with a history of adverse pregnancy outcomes than in women without a history of adverse pregnancy outcomes.

In a meta‐analysis, A Chen et al. [40] evaluated the association of gestational diabetes and the development of overall cardiovascular disease by pooling the results of 38 studies including 77,678,684 participants and showed that GDM increased the risk of developing cardiovascular disease for mothers and newborns by 46% and 23%, respectively. These results indicate the critical role of preventing diabetes and hypertension after GDM in women who have previously had GDM to reduce the risk of vascular and renal disease.

The association between GDM and an increased risk of developing CKD in postpartum women may be explained by potential biological mechanisms involving multiple factors pre‐ and post‐pregnancy, including subclinical inflammatory processes, insulin resistance, beta‐cell failure, and vascular dysfunction influenced by genetic, epigenetic, obesity, lifestyle, and environmental factors [17, 26, 43]. Increased albuminuria among women with a history of GDM compared to women without a history of GDM is one of the hypotheses proposed to explain the association between GDM and early CKD [17, 44]. A number of studies have shown that albuminuria is higher among women with a history of GDM than among women without a history of GDM [17, 45, 46]. Another possible mechanism is the association between GDM as an independent risk factor for subclinical inflammation and endothelial dysfunction. Studies have shown that women with GDM are at higher risk of metabolic syndrome and microvascular complications [25, 47, 48]. We did not find any association between GDM and acute renal failure, which is not surprising and may be due to the limited number of studies in this area. New prospective studies are recommended to explore the association between GDM and acute kidney disease.

### 4.1 Limitation

Our study had limitations and strengths that should be noted. First, we were unable to estimate the effect size of the association of GDM with the risk of developing maternal CKD based on a number of key variables, including preeclampsia, blood pressure, socioeconomic status, and gestational age that may have influenced the study results because they were not reported in the original included studies or were unavailable. Second, these studies were conducted in specific populations with their own characteristics, and generalization of the results to other studies should be done with caution. The assessment of the quantitative association of GDM with maternal CKD after delivery in a comprehensive review was the most important strength of this meta‐analysis.

## 5 Conclusion

GDM with and without DM was significantly associated with an increased risk of developing CKD in postpartum women. DM with GDM had a synergistic effect on the risk of developing maternal CKD, and the risk of developing CKD was significantly higher in GDM women with DM+ than in GDM women with DM‐. The literature review and results of this meta‐analysis suggest that women who experience GDM during their pregnancy, especially those who later develop DM, should be well informed about their long‐term risk of kidney disease. This knowledge is essential for early preventive measures and follow‐up care.

## Author Contributions

**Zahra Aghakhani:** data curation, software, investigation, writing – original draft, writing – review and editing. **Sheida Shokohyar:** conceptualization, writing – review and editing, writing – original draft, methodology, software, data curation. **Elham Rahimpour:** methodology, conceptualization, investigation, funding acquisition, visualization, formal analysis, software, writing – original draft. **Mohammadsadra Shamohammadi:** conceptualization, formal analysis, software, data curation, investigation, writing – original draft, writing – review and editing. **Azin Ghaffari:** conceptualization, investigation, funding acquisition, writing – review and editing, writing – original draft, validation, methodology, software, formal analysis, project administration, data curation, supervision. **Meisam Haghmoradi:** conceptualization, visualization, writing – review and editing, writing – original draft. **Ali Delpisheh:** formal analysis, software, methodology, investigation, writing – original draft, writing – review and editing. **Mansour Bahardoust:** conceptualization, investigation, funding acquisition, writing – original draft, writing – review and editing, methodology, software, formal analysis, data curation, supervision.

## Funding

The authors have nothing to report.

## Ethics Statement

Protocol registered in PROSPERO (registration number: CRD42024604213).

## Conflicts of Interest

The authors declare no conflicts of interest.

## Supporting information


**Figure S1:** Funnel plot for sensitivity analysis.
**Table S1:** Search strategies.
**Table S2:** CKD definition in each study.
**Table S3:** Risk of bias.

## Data Availability

The data that support the findings of this study are available on request from the corresponding author. The data are not publicly available due to privacy or ethical restrictions.

## References

[1] A. Sweeting , W. Hannah , H. Backman , et al., “Epidemiology and Management of Gestational Diabetes,” Lancet 404, no. 10448 (2024): 175–192. 38909620 10.1016/S0140-6736(24)00825-0

[2] E. Vounzoulaki , K. Khunti , S. C. Abner , B. K. Tan , M. J. Davies , and C. L. Gillies , “Progression to Type 2 Diabetes in Women With a Known History of Gestational Diabetes: Systematic Review and Meta‐Analysis,” BMJ 369 (2020): m1361. 32404325 10.1136/bmj.m1361 PMC7218708

[3] N. Malaza , M. Masete , S. Adam , S. Dias , T. Nyawo , and C. Pheiffer , “A Systematic Review to Compare Adverse Pregnancy Outcomes in Women With Pregestational Diabetes and Gestational Diabetes,” International Journal of Environmental Research and Public Health 19, no. 17 (2022): 10846. 36078559 10.3390/ijerph191710846 PMC9517767

[4] A. C. O'Higgins , V. O'Dwyer , C. O'Connor , S. F. Daly , B. T. Kinsley , and M. J. Turner , “Postpartum Dyslipidaemia in Women Diagnosed With Gestational Diabetes Mellitus,” Irish Journal of Medical Science 186, no. 2 (2017): 403–407. 27401735 10.1007/s11845-016-1474-y

[5] M. V. Diaz‐Santana , K. M. O’Brien , Y. M. M. Park , D. P. Sandler , and C. R. Weinberg , “Persistence of Risk for Type 2 Diabetes After Gestational Diabetes Mellitus,” Diabetes Care 45, no. 4 (2022): 864–870. 35104325 10.2337/dc21-1430 PMC9016728

[6] S. M. Lee , M. Shivakumar , J. W. Park , et al., “Long‐Term Cardiovascular Outcomes of Gestational Diabetes Mellitus: A Prospective UK Biobank Study,” Cardiovascular Diabetology 21, no. 1 (2022): 221. 36309714 10.1186/s12933-022-01663-w PMC9618212

[7] M. J. Hare , M. J. L. Hare , L. J. Maple‐Brown , et al., “Risk of Kidney Disease Following a Pregnancy Complicated by Diabetes: A Longitudinal, Population‐Based Data‐Linkage Study Among Aboriginal Women in the Northern Territory, *Australia* ,” Diabetologia 66, no. 5 (2023): 837–846. 36651940 10.1007/s00125-023-05868-w PMC10036460

[8] P. M. Barrett , F. P. McCarthy , K. Kublickiene , et al., “Adverse Pregnancy Outcomes and Long‐Term Maternal Kidney Disease: A Systematic Review and Meta‐Analysis,” JAMA Network Open 3, no. 2 (2020): e1920964. 32049292 10.1001/jamanetworkopen.2019.20964 PMC12527481

[9] P. E. Stevens , S. B. Ahmed , J. J. Carrero , et al., “KDIGO 2024 Clinical Practice Guideline for the Evaluation and Management of Chronic Kidney Disease,” Kidney International 105, no. 4 (2024): S117–S314. 38490803 10.1016/j.kint.2023.10.018

[10] A. S. Minhas , M. Countouris , C. E. Ndumele , et al., “Association of Gestational Diabetes With Subclinical Cardiovascular Disease,” JACC: Advances 3, no. 8 (2024): 101111. 39105123 10.1016/j.jacadv.2024.101111 PMC11299583

[11] D. K. Tobias , F. B. Hu , J. P. Forman , J. Chavarro , and C. Zhang , “Increased Risk of Hypertension After Gestational Diabetes Mellitus: Findings From a Large Prospective Cohort Study,” Diabetes Care 34, no. 7 (2011): 1582–1584. 21593289 10.2337/dc11-0268 PMC3120181

[12] M. F. B. de Resende Guimarães , A. H. F. Brandão , C. A. de Lima Rezende , et al., “Assessment of Endothelial Function in Pregnant Women With Preeclampsia and Gestational Diabetes Mellitus by Flow‐Mediated Dilation of Brachial Artery,” Archives of Gynecology and Obstetrics 290, no. 3 (2014): 441–447. 24691824 10.1007/s00404-014-3220-x

[13] K. Dipla , A. Triantafyllou , I. Grigoriadou , et al., “Impairments in Microvascular Function and Skeletal Muscle Oxygenation in Women With Gestational Diabetes Mellitus: Links to Cardiovascular Disease Risk Factors,” Diabetologia 60, no. 1 (2017): 192–201. 27722775 10.1007/s00125-016-4129-7

[14] S. Rawal , S. F. Olsen , L. G. Grunnet , et al., “Gestational Diabetes Mellitus and Renal Function: A Prospective Study With 9‐ to 16‐Year Follow‐Up After Pregnancy,” Diabetes Care 41, no. 7 (2018): 1378–1384. 29728364 10.2337/dc17-2629 PMC6014536

[15] M. H. Christensen , C. Bistrup , K. H. Rubin , et al., “Kidney Disease in Women With Previous Gestational Diabetes Mellitus: A Nationwide Register‐Based Cohort Study,” Diabetes Care 47, no. 3 (2023): 401–408. 10.2337/dc23-1092 38100751

[16] E. W. Dehmer , M. A. Phadnis , E. P. Gunderson , et al., “Association Between Gestational Diabetes and Incident Maternal CKD: The Coronary Artery Risk Development in Young Adults (CARDIA) Study,” American Journal of Kidney Diseases 71, no. 1 (2018): 112–122. 29128412 10.1053/j.ajkd.2017.08.015 PMC5742081

[17] A. S. Bomback , Y. Rekhtman , A. T. Whaley‐Connell , et al., “Gestational Diabetes Mellitus Alone in the Absence of Subsequent Diabetes Is Associated With Microalbuminuria: Results From the Kidney Early Evaluation Program (KEEP),” Diabetes Care 33, no. 12 (2010): 2586–2591. 20807871 10.2337/dc10-1095 PMC2992195

[18] S.‐T. Tseng , M. C. Lee , Y. T. Tsai , et al., “Risks After Gestational Diabetes Mellitus in Taiwanese Women: A Nationwide Retrospective Cohort Study,” Biomedicine 11, no. 8 (2023): 2120. 10.3390/biomedicines11082120 PMC10452882 37626617

[19] B. M. Daly , Z. Wu , K. Nirantharakumar , L. Chepulis , J. A. Rowan , and R. K. R. Scragg , “Increased Risk of Cardiovascular and Renal Disease, and Diabetes for All Women Diagnosed With Gestational Diabetes Mellitus in New Zealand:A National Retrospective Cohort Study,” Journal of Diabetes 16, no. 4 (2024): e13535. 38599878 10.1111/1753-0407.13535 PMC11006618

[20] A. Backal , S. Vasudevan , R. Lee , E. B. Rosenfeld , and C. V. Ananth , “Pregestational and Gestational Diabetes Mellitus and Risk of Postpartum Kidney Disease: A Retrospective Cohort Study,” Diabetes Research and Clinical Practice 226 (2025): 112330. 40532766 10.1016/j.diabres.2025.112330

[21] R. E. O'Dea , M. Lagisz , M. D. Jennions , et al., “Preferred Reporting Items for Systematic Reviews and Meta‐Analyses in Ecology and Evolutionary Biology: A PRISMA Extension,” Biological Reviews 96, no. 5 (2021): 1695–1722. 33960637 10.1111/brv.12721 PMC8518748

[22] A. Levin , P. E. Stevens , R. W. Bilous , et al., “Kidney Disease: Improving Global Outcomes (KDIGO) CKD Work Group. KDIGO 2012 Clinical Practice Guideline for the Evaluation and Management of Chronic Kidney Disease,” Kidney International. Supplement 3, no. 1 (2013): 1–150.

[23] G. Wells , The Newcastle‐Ottawa Scale (NOS) for Assessing the Quality of Nonrandomised Studies in Meta‐Analyses (Ottawa Hospital Research Institute, 2012).

[24] M. Prasad , “Introduction to the GRADE Tool for Rating Certainty in Evidence and Recommendations,” Clinical Epidemiology and Global Health 25 (2024): 101484.

[25] P. M. Barrett , F. P. McCarthy , M. Evans , et al., “Does Gestational Diabetes Increase the Risk of Maternal Kidney Disease? A Swedish National Cohort Study,” PLoS One 17, no. 3 (2022): e0264992. 35271650 10.1371/journal.pone.0264992 PMC8912264

[26] O. Beharier , I. Shoham‐Vardi , G. Pariente , et al., “Gestational Diabetes Mellitus Is a Significant Risk Factor for Long‐Term Maternal Renal Disease,” Journal of Clinical Endocrinology & Metabolism 100, no. 4 (2015): 1412–1416. 25668200 10.1210/jc.2014-4474

[27] C. Crump , J. Sundquist , and K. Sundquist , “Adverse Pregnancy Outcomes and Long‐Term Risk of Chronic Kidney Disease in Women: National Cohort and Co‐Sibling Study,” American Journal of Obstetrics and Gynecology 230, no. 5 (2024): 563.e1–563.e20. 10.1016/j.ajog.2023.10.008 PMC11006822 37827269

[28] A. K. Bello , B. Hemmelgarn , M. Lin , et al., “Impact of Remote Location on Quality Care Delivery and Relationships to Adverse Health Outcomes in Patients With Diabetes and Chronic Kidney Disease,” Nephrology, Dialysis, Transplantation 27, no. 10 (2012): 3849–3855. 10.1093/ndt/gfs267 22759385

[29] X. S. Feng , R. Farej , B. B. Dean , et al., “CKD Prevalence Among Patients With and Without Type 2 Diabetes: Regional Differences in the United States,” Kidney Medicine 4, no. 1 (2022): 100385. 35072048 10.1016/j.xkme.2021.09.003 PMC8767132

[30] L. R. Zelnick , N. S. Weiss , B. R. Kestenbaum , et al., “Diabetes and CKD in the United States Population, 2009–2014,” Clinical Journal of the American Society of Nephrology 12, no. 12 (2017): 1984–1990. 29054846 10.2215/CJN.03700417 PMC5718269

[31] H. Li , W. Lu , A. Wang , H. Jiang , and J. Lyu , “Changing Epidemiology of Chronic Kidney Disease as a Result of Type 2 Diabetes Mellitus From 1990 to 2017: Estimates From Global Burden of Disease 2017,” Journal of Diabetes Investigation 12, no. 3 (2021): 346–356. 32654341 10.1111/jdi.13355 PMC7926234

[32] E. Figueroa‐Solis , D. Gimeno Ruiz de Porras , M. Rojas‐Garbanzo , L. Whitehead , K. Zhang , and G. L. Delclos , “Prevalence and Geographic Distribution of Self‐Reported Chronic Kidney Disease and Potential Risk Factors in Central America,” International Journal of Environmental Research and Public Health 20, no. 2 (2023): 1308. 36674063 10.3390/ijerph20021308 PMC9859154

[33] T. Yin , Y. Chen , L. Tang , H. Yuan , X. Zeng , and P. Fu , “Relationship Between Modifiable Lifestyle Factors and Chronic Kidney Disease: A Bibliometric Analysis of Top‐Cited Publications From 2011 to 2020,” BMC Nephrology 23, no. 1 (2022): 120. 35337272 10.1186/s12882-022-02745-3 PMC8957172

[34] K.‐H. Mak , E. Vidal‐Petiot , R. Young , et al., “Prevalence of Diabetes and Impact on Cardiovascular Events and Mortality in Patients With Chronic Coronary Syndromes, Across Multiple Geographical Regions and Ethnicities,” European Journal of Preventive Cardiology 28, no. 16 (2021): 1795–1806. 10.1093/eurjpc/zwab011 35022686

[35] E. K. Tannor , O. U. Chika , and I. G. Okpechi , “The Impact of Low Socioeconomic Status on Progression of Chronic Kidney Disease in Low‐and Lower Middle‐Income Countries,” Seminars in Nephrology 42, no. 5 (2022): 151338, 10.1016/j.semnephrol.2023.151338. 36966563

[36] R. E. Patzer and W. M. McClellan , “Influence of Race, Ethnicity and Socioeconomic Status on Kidney Disease,” Nature Reviews Nephrology 8, no. 9 (2012): 533–541. 22735764 10.1038/nrneph.2012.117 PMC3950900

[37] A. M. Hazara and S. Bhandari , “Age, Gender and Diabetes as Risk Factors for Early Mortality in Dialysis Patients: A Systematic Review,” Clinical Medicine & Research 19, no. 2 (2021): 54–63. 33582647 10.3121/cmr.2020.1541 PMC8231690

[38] Y. Deng , N. Li , Y. Wu , et al., “Global, Regional, and National Burden of Diabetes‐Related Chronic Kidney Disease From 1990 to 2019,” Frontiers in Endocrinology 12 (2021): 672350. 34276558 10.3389/fendo.2021.672350 PMC8281340

[39] X.‐Q. Wu , D. D. Zhang , Y. N. Wang , Y. Q. Tan , X. Y. Yu , and Y. Y. Zhao , “AGE/RAGE in Diabetic Kidney Disease and Ageing Kidney,” Free Radical Biology and Medicine 171 (2021): 260–271. 34019934 10.1016/j.freeradbiomed.2021.05.025

[40] A. Chen , B. Tan , R. du , et al., “Gestational Diabetes Mellitus and Development of Intergenerational Overall and Subtypes of Cardiovascular Diseases: A Systematic Review and Meta‐Analysis,” Cardiovascular Diabetology 23, no. 1 (2024): 320. 39198842 10.1186/s12933-024-02416-7 PMC11360578

[41] K. Siddiqui and T. P. George , “The Potential Impact of Gestational Diabetes Mellitus on Long‐Term Kidney Disease: A Narrative Review,” EMJ Diabetes 12, no. 1 (2024): 57–64, 10.33590/emjdiabet/GPTZ1914.

[42] R. Neiger , “Long‐Term Effects of Pregnancy Complications on Maternal Health: A Review,” Journal of Clinical Medicine 6, no. 8 (2017): 76. 28749442 10.3390/jcm6080076 PMC5575578

[43] H. D. McIntyre , J. Fuglsang , U. Kampmann , S. Knorr , and P. Ovesen , “Hyperglycemia in Pregnancy and Women's Health in the 21st Century,” International Journal of Environmental Research and Public Health 19, no. 24 (2022): 16827. 36554709 10.3390/ijerph192416827 PMC9779688

[44] S. Friedman , D. Rabinerson , J. Bar , et al., “Microalbuminuria Following Gestational Diabetes,” Acta Obstetricia et Gynecologica Scandinavica 74, no. 5 (1995): 356–360. 7778427 10.3109/00016349509024428

[45] C. Kim , Y. J. Cheng , and G. L. Beckles , “Cardiovascular Disease Risk Profiles in Women With Histories of Gestational Diabetes but Without Current Diabetes,” Obstetrics & Gynecology 112, no. 4 (2008): 875–883. 18827131 10.1097/AOG.0b013e31818638b5 PMC2610423

[46] M. Dallatu , A. M. Kaoje , A. U. Adoke , J. A. Kehinde , and J. M. Bunza , “Microalbuminuria in Women With Risk Factors for Gestational Diabetes Mellitus in Some Selected Hospitals in Sokoto, Nigeria,” Asian Journal of Research in Medical and Pharmaceutical Sciences 6 (2019): 1–8.

[47] R. Retnakaran and B. R. Shah , “Role of Type 2 Diabetes in Determining Retinal, Renal, and Cardiovascular Outcomes in Women With Previous Gestational Diabetes Mellitus,” Diabetes Care 40, no. 1 (2017): 101–108. 27821407 10.2337/dc16-1400

[48] M. Lappas , “Markers of Endothelial Cell Dysfunction Are Increased in Human Omental Adipose Tissue From Women With Pre‐Existing Maternal Obesity and Gestational Diabetes,” Metabolism 63, no. 6 (2014): 860–873. 24684825 10.1016/j.metabol.2014.03.007

