# Supplementary material for: Gestational Diabetes and Long‐Term Risk of Maternal Kidney Disease: Systematic Review and Meta‐Analysis of Population Base Cohort Studies
Source: Endocrinol Diabetes Metab. 2026 Apr 22;9(3):e70208. doi: 10.1002/edm2.70208 (PMC13101955; doi:10.1002/edm2.70208)
Supplement: Supplementary file 1 — Figure S1: Funnel plot for sensitivity analysis. Table S1: Search strategies. Table S2: CKD definition in each study. Table S3: Risk of bias. [file EDM2-9-e70208-s001.docx]

Table 1: Search strategies for searching databases.

| Data Base | **Search Strategy** |
| --- | --- |
| **PubMed** | (((Gestational Diabetes Mellitus[MeSH Terms] OR Gestational Diabetes Mellitus[Title/Abstract] OR Gestational diabetes[MeSH Terms] OR Gestational diabetes[All Fields] OR Gestational Diabetes[Title/Abstract] OR Gestational Diabetes[All Fields] OR Pregnancy-Induced Diabetes[All Fields] OR Pregnancy-Induced Diabetes[Title/Abstract]) AND (chronic kidney disease[Title/Abstract] OR chronic kidney disease [All Fields] OR kidney disease [All Fields] OR Chronic Renal Disease [All Fields] OR Chronic Renal Disease [Title/Abstract]) AND (Women[MeSH Terms] OR women [Title/Abstract] OR maternal [Title/Abstract] OR maternal [Title/Abstract]))) |
| Scopus | ( TITLE-ABS-KEY (Gestational Diabetes Mellitus ) OR TITLE-ABS-KEY (Gestational Diabetes) OR TITLE-ABS-KEY (Pregnancy-Induced Diabetes)) ) AND ( TITLE-ABS-KEY ( chronic kidney disease ) OR TITLE-ABS-KEY (kidney disease) OR TITLE-ABS-KEY ( Maternal chronic kidney disease) OR TITLE-ABS-KEY ( Chronic Renal Disease)) ) AND TITLE-ABS-KEY ( women OR maternal) |
| Web of Science | Maternal OR women (Topic) AND Gestational Diabetes Mellitus OR Gestational Diabetes OR (Pregnancy-Induced Diabetes) (Topic) AND chronic kidney disease OR chronic kidney failure OR (Chronic Renal Disease) OR maternal chronic kidney disease OR (renal disease). |

Table 2: CKD definition in each studies.

| Authors (Year) | **CKD definition** |
| --- | --- |
| AS Bomback(2010)[18] | eGFR 30–59 ml/min per 1.73 m2 or eGFR <30mL/min/1.73m2 and end-stage kidney disease (ESKD) (i.e. stage 4–5 CKD) |
| O Beharier(2015)[25] | ICD10: Acute and chronic kidney disease ( N17 and N18) |
| S Rawal(2018)[26] | eGFR <90 ml/min per 1.73 m2 |
| EW Dehmer(2018)[27] | eGFR 30–59 ml/min per 1.73 m2 |
| PM Barrett(2022)[28] | end-stage kidney disease (ESKD) : eGFR <30mL/min/1.73m2 (i.e. stage 4–5 CKD) |
| ST Tseng(2023)[19] | ICD10: chronic kidney disease ( N18)  ICD10:chronic kidney disease ( 585) |
| MJL Hare(2023) [7] | Any CKD incorporated ICD-10-AM codes for any stage of CKD or dialysis.  Stages 1-5 CKD (2009-2018)  End stage renal disease (2000-2008)  Other chronic renal failure (2000-2008)  Unspecified CKD (2000-2018)  Dependence on renal dialysis (2000-2018)  Preparatory care for dialysis (2000-2018)  Extracorporeal dialysis (2000-2018) |
| MH Christensen(2024) [20] | eGFR <30mL/min/1.73m2 (i.e. stage 4–5 CKD) |
| BM Daly(2024) [21] | ICD10: Acute and chronic kidney disease ( N17 and N18) |
| C Crump(2024) [29] | ICD10: chronic kidney disease ( N18)  ICD10: chronic kidney disease ( 585) |
| A Backal(2025) [22] | ICD10: chronic kidney disease ( N18)  ICD10: chronic kidney disease ( 585) |

**Table 3: Evaluation of the quality of studies based on the checklist Newcastle-Ottawa**

| Authors (Year) | **Selection** | **Comparability** | **Outcome** | **Classification based on AHRQ standards** |
| --- | --- | --- | --- | --- |
| AS Bomback(2010)[1] | *** | ** | *** | Good |
| O Beharier(2015)[2] | **** | ** | *** | Good |
| S Rawal(2018)[3] | *** | ** | *** | Good |
| EW Dehmer(2018)[4] | *** | ** | ** | Good |
| PM Barrett(2022)[5] | **** | * | ** | Good |
| ST Tseng(2023)[6] | **** | ** | *** | Good |
| MJL Hare(2023)[7] | ** | * | ** | Fair |
| MH Christensen(2024)[8] | **** | ** | ** | Good |
| BM Daly(2024)[9] | *** | ** | ** | Good |
| C Crump(2024)[10] | *** | ** | ** | Good |
| A Backal(2025)[11] | **** | ** | *** | Good |


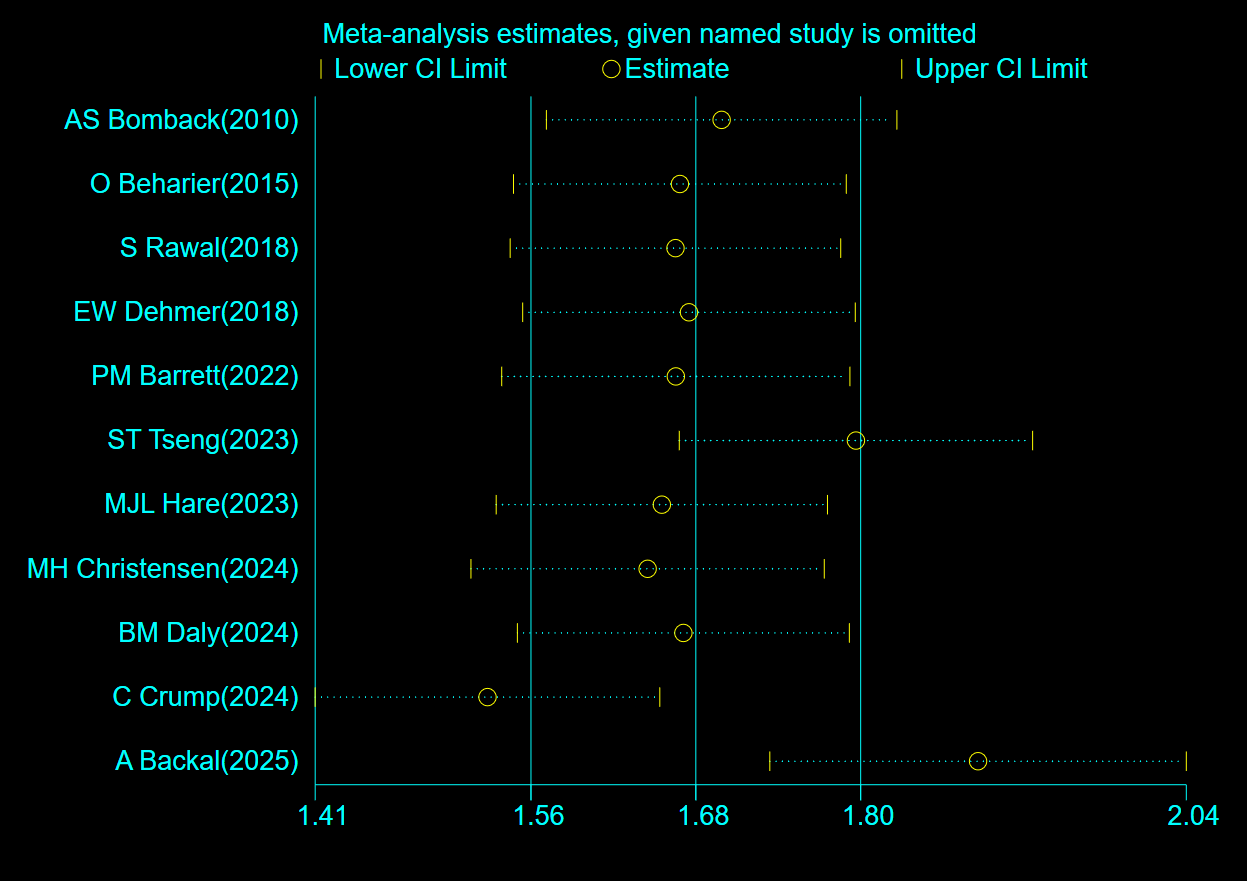


Figure 1: The effect of individual studies on the overall estimate of the association of GDM with maternal CKD based on sensitivity analysis

1. Bomback, A.S., et al., *Gestational diabetes mellitus alone in the absence of subsequent diabetes is associated with microalbuminuria: results from the Kidney Early Evaluation Program (KEEP).* Diabetes care, 2010. **33**(12): p. 2586-2591.

2. Beharier, O., et al., *Gestational diabetes mellitus is a significant risk factor for long-term maternal renal disease.* The Journal of Clinical Endocrinology & Metabolism, 2015. **100**(4): p. 1412-1416.

3. Rawal, S., et al., *Gestational diabetes mellitus and renal function: a prospective study with 9-to 16-year follow-up after pregnancy.* Diabetes care, 2018. **41**(7): p. 1378-1384.

4. Dehmer, E.W., et al., *Association between gestational diabetes and incident maternal CKD: the Coronary Artery Risk Development in Young Adults (CARDIA) study.* American Journal of Kidney Diseases, 2018. **71**(1): p. 112-122.

5. Barrett, P.M., et al., *Does gestational diabetes increase the risk of maternal kidney disease? A Swedish national cohort study.* PLoS One, 2022. **17**(3): p. e0264992.

6. Tseng, S.-T., et al., *Risks after gestational diabetes mellitus in Taiwanese women: a nationwide retrospective cohort study.* Biomedicines, 2023. **11**(8): p. 2120.

7. Hare, M.J., et al., *Risk of kidney disease following a pregnancy complicated by diabetes: a longitudinal, population-based data-linkage study among Aboriginal women in the Northern Territory, Australia.* Diabetologia, 2023. **66**(5): p. 837-846.

8. Christensen, M.H., et al., *Kidney disease in women with previous gestational diabetes mellitus: a nationwide register-based cohort study.* Diabetes Care, 2024. **47**(3): p. 401-408.

9. Daly, B.M., et al., *Increased risk of cardiovascular and renal disease, and diabetes for all women diagnosed with gestational diabetes mellitus in New Zealand—A national retrospective cohort study.* Journal of diabetes, 2024. **16**(4): p. e13535.

10. Crump, C., J. Sundquist, and K. Sundquist, *Adverse pregnancy outcomes and long-term risk of chronic kidney disease in women: national cohort and co-sibling study.* American journal of obstetrics and gynecology, 2024. **230**(5): p. 563. e1-563. e20.

11. Backal, A., et al., *Pregestational and gestational diabetes mellitus and risk of postpartum kidney disease: A retrospective cohort study.* Diabetes Research and Clinical Practice, 2025: p. 112330.
